# Reduced Neuromuscular Performance in Night Shift Orthopedic Nurses: New Insights From a Combined Electromyographic and Force Signals Approach

**DOI:** 10.3389/fphys.2020.00693

**Published:** 2020-06-30

**Authors:** Emiliano Cè, Christian Doria, Eliana Roveda, Angela Montaruli, Letizia Galasso, Lucia Castelli, Antonino Mulè, Stefano Longo, Giuseppe Coratella, Pasqualino D’Aloia, Giuseppe Banfi, Fabio Esposito

**Affiliations:** ^1^Department of Biomedical Sciences for Health, Università Degli Studi di Milano, Milan, Italy; ^2^IRCCS Istituto Ortopedico Galeazzi, Milan, Italy

**Keywords:** night shift workers, nurse, fatigue, neuromuscular control, activity level, sleep-wake rhythm

## Abstract

The effect of sleep–wake rhythm disruption on neuromuscular control and muscle fatigue has received little attention. Because nurse shift work is so varied, including overnight duty, rotating shift schedules, early awakening, and interrupted nocturnal sleep, it offers an interesting model to study this paradigm. It has been investigated so far using only subjective markers. A combined approach based on the simultaneous analysis of surface electromyographic (sEMG) and force signals can objectively detect possible deficits in neuromuscular control and muscle fatigue. With this study we investigated neuromuscular activation and muscle contraction capacity at submaximum and maximum level in nurses working two night-shift schedules and compared them to levels in nurses working entirely in day shifts. Sleep quality and activity levels were also assessed. The study sample was 71 nurses grouped by their shift work schedule: night shift for 5 days (NS_5_, *n* = 46), night shift for 10 days (NS_10_, *n* = 9), and only day/swing shift (DS, *n* = 16). Before and after the shift-work cycle, maximum voluntary contraction (MVC) force and muscle activation, neuromuscular control, and muscle fatigability were measured in the finger flexor muscles. Activity level and sleep quality during the shift-work cycle were recorded with a wrist actigraph. After the shift-work cycles, MVC force and muscle activation were decreased (−11 ± 3% and −33 ± 3%, *p* < 0.001) as was neuromuscular control (−36 ± 8%, *p* = 0.007), whereas muscle fatigability was increased (+ 19 ± 9%, *p* = 0.006) in the NS_5_ and the NS_10_ group. Sleep quality was lower in the NS_5_ and the NS_10_ group (−8 ± 1.8% and −15%3, respectively, *p* < 0.001), while the activity level for the three groups was similar. There was a clear reduction in neuromuscular control and an increase in muscle fatigue in the nurses working the night shift. These findings may inform of work schedule planning or recommendations for devising new recovery strategies to counteract neuromuscular alterations in night shift nurses.

## Introduction

Hospital organizations operate around the clock and so must rotate staff between day shifts and night shifts. But because night-shift workers routinely work against their internal biological clock, they can suffer from sleepiness caused by desynchronization of their internal circadian clock and disturbance in their sleep-wake pattern. Nursing staff work shifts to provide continuous 24 h care (Patterson et al., 2010; Yuan et al., 2011; Bae and Fabry, 2014). Nurse shift work entails overnight duty, rotating schedules, early awakening, and interrupted nocturnal sleep. These alternating work schedules disrupt circadian rhythms and result in a mismatch between a nurse’s individual circadian phase and her/his work-cycle schedule (Kuhn, 2001).

A leading cause of shift-work disorders is the decrease in sleep quantity and quality. Many biological functions besides sleep are regulated by circadian rhythms and their expression can differ across individuals. Circadian preferences are governed by internal factors (e.g., clock genes, cortisol, and melatonin levels) and environmental factors (e.g., social habits, light/darkness cycle, season) (Montaruli et al., 2017). In humans, the circadian system consists of multiple oscillators distributed throughout the organism and a central pacemaker in the suprachiasmatic nucleus of the hypothalamus that coordinates the entire system. Chronic disruption of circadian rhythm and sleep is associated with a variety of health risks, including sleep disorders, gastrointestinal disturbances, and cardiovascular disease (Moore-Ede, 1985; Smith and Eastman, 2012; Vitale et al., 2018). The loss of synchronization with the environment disrupts the sleep-wake cycle and the circadian rhythm of locomotor activity (Roveda et al., 2018, 2019; Galasso et al., 2019). Such health risks are often compounded by a reduction in adequate levels of activity (Vitale et al., 2018; Min et al., 2019). Indeed, working the night shift makes it difficult to engage in non-work activities and/or manage social obligations (Sack et al., 2007). Furthermore, night-shift nursing staff is particularly susceptible to circadian misalignment because they sleep during the day, out of phase with their intrinsic circadian sleep–wake rhythm (Yuan et al., 2011; Min et al., 2019). Recovery of lost night-time sleep with daytime sleep is inefficient because of circadian misalignment and it may result in shorter overall sleep duration (Yuan et al., 2011; Min et al., 2019). Partial sleep deprivation impairs central nervous system activities from basic functions of appetite and temperature regulation to higher functions of cognition and vigilance (Steele et al., 1999; Horwitz and McCall, 2004; Barger et al., 2005). Moreover, higher fatigue perception is often reported by nurses working night shifts (Yuan et al., 2011; Min et al., 2019). This has been related to increased occurrence of unintentional incidents, such as on-the-job injuries, and increased health risk for nurses and patients alike (Steele et al., 1999; Horwitz and McCall, 2004; Barger et al., 2005). Despite the high incidence of fatigue and occupational injuries among night shift nurses (Yuan et al., 2011; Min et al., 2019), studies investigating shift work fatigue perception have used only subjective markers obtained from questionnaires that provide limited mechanistic insights into the possible mechanisms underlying this phenomenon (Yuan et al., 2011; Min et al., 2019).

Literature exists on the relationship between sleep restriction/deprivation, muscle force generating capacity and fatigue, with often conflicting findings: on one hand, no changes in maximum force were retrieved after one night of sleep deprivation (Skurvydas et al., 2020), on the other hand, reduction in submaximal and maximal weight-lifting capacity was reported after a period of sleep restriction (Reilly and Piercy, 1994; Arnal et al., 2016). Moreover, the exact mechanisms underlying the possible effect of sleep restriction/deprivation on muscle force generating capacity and fatigue are still under debate (Fullagar et al., 2015; Arnal et al., 2016). Some studies, though, were conducted by simulating sleep deprivation/restriction in a controlled laboratory contest, which may not be representative of what really happens in a real-life/working context. Moreover, some investigating approaches are not easily feasible in a real-work condition (Arnal et al., 2016). To the best of our knowledge, only two studies have used objective physiologic parameters to describe in an “ecological context” fatigue occurrence in nurses (Yuan et al., 2011; Thompson, 2019). Both reported a decrease in muscle force in nurses after having worked the night shift (Yuan et al., 2011; Thompson, 2019). The decrease was higher after three 12 h night shifts within a 72 h period (Thompson, 2019). The studies provided no mechanistic support to better understand fatigue in night-shift workers, reported conflicting results on handgrip muscles, and did not measure sleep quality. Differently, an approach based on the simultaneous analysis of surface electromyographic (sEMG) and force signals may be useful in objectively detecting fatigue and in evaluating deficits in neuromuscular control (Cè et al., 2019). This combined non-invasive approach is feasible in an ecological context and compatible with a hospital environment.

With this in mind, we investigated neuromuscular activation and submaximum and maximum muscle contraction capacity in nurses working two different night-shift schedules and compared them to data from nurses working entirely day shifts. Sleep quality and activity levels were also assessed. Our hypothesis was that we would find larger deficits in force generation capacity and in neuromuscular control possibly associated with poor sleep quality in the nurses working night shifts.

## Materials and Methods

### Participants

The study involved the nursing staff of the Galeazzi Orthopedic Institute (Milan, Italy). The inclusion criterion was continuity of shift work cycle for at least 1 year. Exclusion criteria were: no present cardiovascular, endocrine, and neuromuscular disease, no pharmacological therapy potentially affecting sleep quality, and no pregnancy (self-reported). Ninety-one of the potentially eligible 120 nurses met the study criteria. After receiving a full explanation of the scope of the study and the possible cost/benefit ratio, 71 nurses provided written informed consent to participate in the study. They were free to withdraw from the study at any time. Table 1 presents the anthropometric characteristics of the study sample. The study was approved by the Ethical Committee of San Raffaele Hospital (CE: 156/int/2017), registered at ClinicalTrials.gov (registration number: NCT03453398), and conducted in accordance with the ethical standards of the latest Helsinki Declaration.

**TABLE 1:** Anthropometric characteristics of the participants.

Characteristic	NS_5_	NS_10_	DS	One-way ANOVA
		
	[Mean (*SD*)]	[Mean (*SD*)]	[Mean (*SD*)]	
No. (F/M)	46 (37/9)	9 (6/3)	16 (13/3)	–
Age (years)	47 (12)	50 (11)	40 (13)	*F* = 2.60, *p* = 0.08
Height (m)	1.65 (0.03)	1.67 (0.04)	1.62 (0.04)*	*F* = 7.36, *p* = 0.001
Body mass (kg)	65 (10)	68 (12)	60 (8)	*F* = 2.27, *p* = 0.11
Body-mass index (kg.m^–2^)	23.9 (4.7)	24.4 (3.1)	22.9 (2.2)	*F* = 0.49, *p* = 0.62

*NS_5_ 5-day night shift; NS_10_ 10-day night shift; DS day/swing shift. ^∗^p < 0.05.*

### Study Design

**FIGURE 1:**
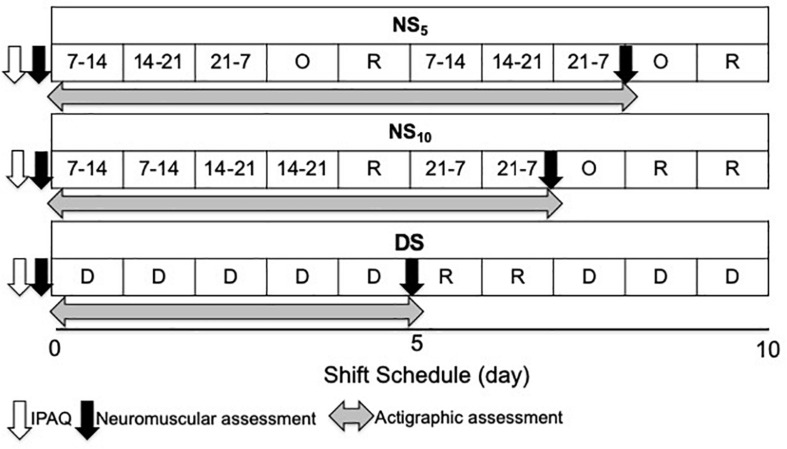
Study flow chart. NS_5_, 5-day night shift; NS_10_, 10-day night shift; DS, day/swing shift; O, night off; R, rest; D, day.

Three groups were formed according to shift work cycle schedule (Figure 1): 24 h shift, with shifts changing every day [from 7.00 to 14.00, from 14.00 to 21.00, from 21.00 to 7.00, 1 night off, and 1 day of rest (5-day night shift cycle, NS_5_)]; 24 h shift, with shifts changing every 2 days [from 7.00 to 14.00 for 2 days, from 14.00 to 21.00 for 2 days, 1 day of rest, from 21.00 to 7.00 for 2 days, 1 night off, 2 days of rest (10-day night shift, NS_10_)]; and only day/swing shift (DS) for 5 days (from 7.00 to 14.00 on 1 day and from 14.00 to 21.00 the next day) followed by 2 days of rest. A total of 46 participants composed the NS_5_, 9 the NS_10_, and 16 the DS group (Table 1).

During the familiarization session the participants practiced with the instrumentation and the experimental set-up and completed the Italian version of the International Physical Activity Questionnaire Short form (IPAQ SF) for assessment of physical activity (Minetto et al., 2018).

On a different day, maximum and submaximal force tests were conducted immediately before the participants entered their work-shift cycle (shift from 7.00 to 14.00 for all three groups, with at least 2 previous days of rest) and after the NS_5_ and the NS_10_ group had worked for 48 h equally divided in morning, swing, and night shifts. Differently, the DS group worked entirely in morning and swing shifts. All participants underwent actigraphic monitoring to assess their physical activity level and sleep quality. They wore a wrist actigraph the day of the first force test and removed it the day of the second force test, i.e., at the end of the work shift. They were instructed to wear the actigraph on the non-dominant arm all day and to remove it only when bathing, swimming, or engaged in combat sports.

During the monitoring period, the participants compiled a sleep diary in which they recorded the hours they went to bed, fell asleep, arose, and any time intervals during which they were not wearing the actigraph. During monitoring, they carried out their routine work and maintained their usual daily activity and their habitual food and beverage intake. In addition, we allowed participants to consume caffeinated or other energetic drinks not to alter their habitual behavior, especially during night work.

### Assessment and Data Analysis

Assessment of physical activity. The Italian version of the IPAQ questionnaire comprises four sets of items that investigate four different aspects of the level of physical activity in relation to health. The participants were asked to record the total amount of time (min) per week they spent performing activities of high and moderate intensity, walking, and sitting. The minutes devoted to each activity were multiplied by a coefficient expressed as metabolic equivalents (MET): METs < 700 indicates *inactive*, METs 701-2519 *sufficiently active*, and METs > 2520 *active*.

**FIGURE 2:**
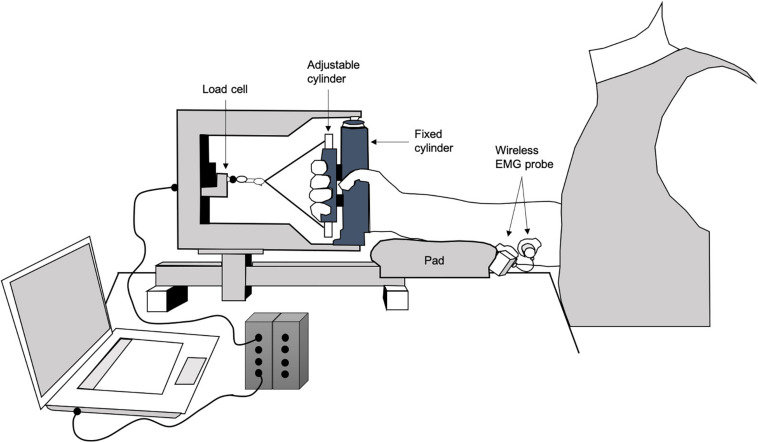
Experimental set up depiction.

Maximum and submaximal force tests. Force and surface electromyographic (sEMG) signals were recorded during isometric contractions from the finger flexor muscles of the dominant arm. The force tests were conducted on a purposely designed ergometer (Limonta et al., 2015) consisting of two cylinders, one fixed and connected to a load cell (Interface, SM- 2000 N, Scottsdale, AZ, United States, linear from 0 to 2,000 N), and the other connected to the fingers at an adjustable distance from the other cylinder (Esposito et al., 2009). The distance between the cylinders was set so that each participant could exert her/his most forceful hand grip. The hand was positioned with the fixed cylinder between the thumb and the second finger, and the adjustable cylinder positioned and blocked at the level of the second finger phalanges. The forearm was kept midway between pronation and supination, with the elbow flexed 90 degrees on a fixed support. The force signal was displayed on a personal computer screen together with the target force to provide visual feedback to the participants (Figure 2). After the monitoring period, maximum voluntary contraction (MVC) force was reassessed and the percentages of the submaximal sustained contractions were calculated accordingly. This allowed to maintain the same relative contraction intensity level between pre and post-measurements (Cè et al., 2013), as it has been found that the percentage MVC force is the determinant of the recruitment level and firing rate of the active motor units (Freund, 1983).

The sEMG signal was detected on the finger flexor muscles (*flexor digitorum superficialis* and *profundus*) by two round Ag/AgCl electrodes with solid hydrogel (mod H124SG Kendall ARBO; diameter 10 mm; interelectrode distance 20 mm; Covidien, Dublin, Ireland). The electrodes were fixed to a probe (probe mass 8.5 g; BTS Inc., Milan, Italy) that wirelessly transmitted the sEMG signal from the subject to the acquisition base (FREEEMG 300, BTS Inc.). The skin area under the sEMG electrodes was shaved, cleaned with ethyl alcohol, abraded gently with fine sandpaper, and prepared with a conductive cream (Nuprep^®^, Weaver and Co., Aurora, CO, United States) to achieve an interelectrode impedance < 2000 Ω. Following the European Recommendations for Surface Electromyography (Hermens et al., 2000), the electrodes were centered around the 50% point on the line joining the medial epicondyle to the styloid process of the ulna and placed between the tendon and the motor point in the direction of the muscle fibers. The sEMG signal was acquired at 1000 Hz, amplified (gain 2000, impedance and the common rejection mode ratio of the equipment > 1015 Ω//0.2 pF and 60/10 Hz 92 dB, respectively) and transmitted to a wireless electromyographic system that digitized (1000 Hz) and filtered (band-pass 10–500 Hz) the raw sEMG signals.

The participants performed three MVC force, each lasting 5 s, with 3 min of rest in between. The highest value was considered the closest to the MVC force. Four 20-s isometric contractions at 20, 40, 60, and 80% MVC force were performed in randomized order. At least 5 min of rest were allowed between two consecutive contractions. To avoid interference from transient phenomena in the submaximum contractions, the 20 s started when the force reached the target force. The participants were then asked to maintain the force output constant (within ± 5%) at all levels of effort with the aid of the visual feedback (target force displayed on the computer screen). Force and sEMG signals were recorded during each contraction and synchronized via a digital push-button. On measurements completion, the sEMG electrodes position was marked on a transparency sheet, together with some skin landmarks (moles, scars, angiomas, etc.) to minimize site-to-site variability in sEMG signals during repeated measurements on different days, permitting a similar repositioning of the electrodes, which were placed by the same experienced operator. This approach was shown to provide very high reliability in sEMG data (Cè et al., 2013; Coratella et al., 2019).

The sEMG signal was analyzed in time and frequency domain: the root mean square (RMS) and the mean frequency (MF) were then calculated in the central 1 s of the MVC force, in the middle of the force plateau. MF of the sEMG signal was calculated by means of the Fast Fourier Transform approach, which was implemented in the sEMG signal acquisition software. sEMG RMS, and MF were calculated in continuous 250 ms time windows during submaximal contractions. The values were normalized for the sEMG RMS and MF recorded during the first s of contraction. The temporal trend of the two parameters was used as a marker to monitor possible changes in neuromuscular activation level of the finger flexors muscles. In particular, the changes over time of the sEMG RMS and MF at 80%MVC force were used as markers of myoelectric fatigue manifestation (Merletti et al., 1990).

The force signal was analyzed in the same time windows as the EMG variables: the force signal coefficient of variation (CV) was estimated by calculating the ratio (expressed as a percentage) between the standard deviation of the force samples and their average in the time window. The distance of the force signal from the target (DT) was calculated in the same time windows as CV. DT was expressed as a percentage to permit the comparison of these variables among participants. Negative DT values described a force output lower than force target and *vice versa.* CV and DT over time provided an index of muscle contraction stability and accuracy, respectively (Limonta et al., 2015). The stability and accuracy of hand muscles are critical to ensure a proper execution of some essential nurses’ tasks.

Assessment of activity levels and sleep quality. An actigraph equipped with a triaxial accelerometer (mod. Motion Watch 8 CamNtech, Cambridge, United Kingdom) was used to record activity levels and sleep quality parameters. The Motion Ware software 1.2.28 (CamNtech) was used to process the activity data, expressed in activity counts, and recorded every 30 s throughout the monitoring period. The data were processed to measure the activity levels of each participant.

The software for sleep analysis (mod. Motion Ware 1.2.28, CamNtech) allowed us to extrapolate six parameters indicative of sleep quantity and quality: (i) *Time in bed*, total time spent in bed between bed time and get up time; (ii) *Assumed sleep time*, time between the beginning and the end of sleep; (iii) *Actual sleep time*, the amount of time between sleep Start and sleep End; this is determined by summation of the number of epochs below the sensitivity threshold and then multiplying that value by the epoch length in min; (iv) *Sleep Efficiency*, the percentage of time in bed actually spent sleeping; (iv) *Sleep Latency*, the amount of time required for sleep onset after retiring to bed; the period between bed time and sleep start is automatically calculated by an algorithm based on lack of movement; (v) *Movement and Fragmentation Index*, the addition of the movement index (% time spent moving) and fragmentation index (% of immobile phases lasting 1 min). The *Movement and Fragmentation Index* was used as an index of restlessness. For all these parameters, an average of the entire monitoring period was calculated for each participant. Moreover, a CR-10 scale (0 = no tired at all, 10 = maximally tired) was used to assess the level of perceived tiredness in all the participants, before and after the monitoring period.

### Statistical Analysis

Statistical analysis was performed using a statistical software package (IBM- SPSS Statistics 25, Armonk, NY, United States). The Shapiro-Wilk test was applied to check normal distribution of the sampling. The between-group differences in the level of daily activity and sleep quality parameters were calculated by one-way analysis of variance (ANOVA) or by equivalent non-parametric Kruskal-Wallis one-way ANOVA on the ranks test if the sample data did not have a normal distribution. The differences in MVC force, sEMG RMS, sEMG MF, and CR-10 scale before and after the monitoring period were calculated for the three groups (NS_5_, NS_10_, and DS) using two-way repeated-measure ANOVA. Three-way repeated-measure ANOVA was used to calculate the differences over time (20 s contraction) in CV, DF, sEMG RMS, and sEMG MF between the three groups, before and after monitoring. Multiple comparisons were performed using Bonferroni’s correction. Significance was set at a *p* < 0.05. Changes are reported as a percentage change with 95% of confidence interval (95% CI). Cohen’s *d* effect size (ES) was calculated and interpreted as follows: *trivial* 0.00–0.19; *small* 0.20–0.59; *moderate* 0.60–1.19; *large* 1.20–1.99; *very large* ≥ 2.00 (Hopkins et al., 2009). The 95% CI of the ES is also reported. Pearson correlation or Spearman correlation tests were applied to check for possible correlations between the percentage changes in MVC force, sEMG RMS and sEMG MF and activity level and sleep quality parameters. Possible correlations between age and the percentage changes in MVC force, sEMG RMS, sEMG MF, activity level and sleep quality parameters were also checked. These latter were tested splitting female from male participants. The magnitude of correlations was interpreted as follows: *trivial* (*R*) < 0.1; *low* 0.10–0.30*; moderate* 0.31–0.50; *high* 0.51–0.70; *very high* 0.71–0.90; *nearly perfect* 0.91–0.99; and *perfect* for *R* = 1 (Hopkins, 2000). Unless otherwise stated, descriptive statistics are presented as mean ± standard deviation (SD).

## Results

Level of physical activity (IPAQ SF questionnaire responses). ANOVA revealed no differences between the groups for the physical activity level assessed before the work shifts [NS_5_ = 5219 (887) METS/w, NS_10_ = 4450 (1069) METS/w, DS = 5526 (1245) METS/w, *F* = 0.10, *p* = 0.903]. In detail, 26 were classified active, 12 sufficiently active, and 8 inactive in the NS_5_ group; 6 were classified active, 3 sufficiently active, and 0 inactive in the NS_10_ group; and 11 were classified active, 4 sufficiently active, and 1 inactive in the DS group.

**FIGURE 3:**
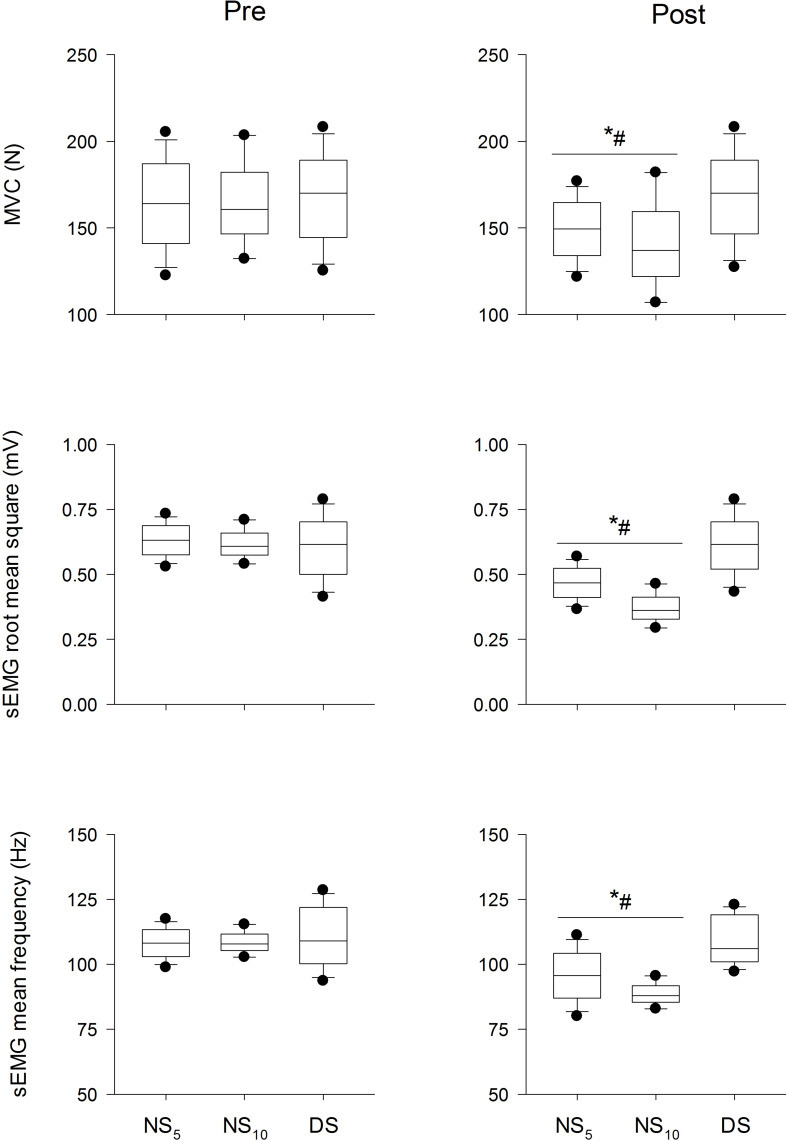
Maximum voluntary contraction force (MVC, upper panel), surface electromyogram root mean square (sEMG RMS, middle panel), and mean frequency (sEMG MF, lower panel) at the beginning (pre) and at the end (post) of the shift cycle for the three groups. NS_5_, 5-day night shift; NS_10_, 10-day night shift; DS, day/swing shift. *p < 0.05 post vs. pre; ^#^*p* < 0.05 NS_5_ or NS_10_ vs. DS. Black dots represent the 95th/5th percentile.

Maximum and submaximum contraction. Figure 3 presents the between-group differences for MVC force (upper panel), sEMG RMS (middle panel), and sEMG MF (lower panel). ANOVA revealed a time x group interaction for MVC force (*F* = 13.74, *p* < 0.001), sEMG RMS (*F* = 11.52, *p* < 0.001), and sEMG MF (*F* = 8.81, *p* < 0.001). *Post hoc* analysis revealed a reduction in MVC force, sEMG RMS and sEMG MF between NS_5_ and NS_10_ vs. DS: MVC force [−21.7 N (−37.2/−6.3), *d* = −1.67 (−2.33/−1.02), and −27.6 N (−49.6/−5.6), *d* = −2.32 (−3.38/−1.26)], sEMG RMS [−63.2 mV (−108.0/−18.4), *d* = −2.34 (−3.06/−1.62), and −116.3 m5 V (−179.9/−57.6), *d* = −4.51 (−6.03/−2.99)], and sEMG MF [−8.5 Hz (−14.4/−2.5), *d* = −1.43 (−2.07/−0.80), and −11.6 Hz (−20.1/−3.2), *d* = −2.57 (−3.68/−1.47)].

**FIGURE 4:**
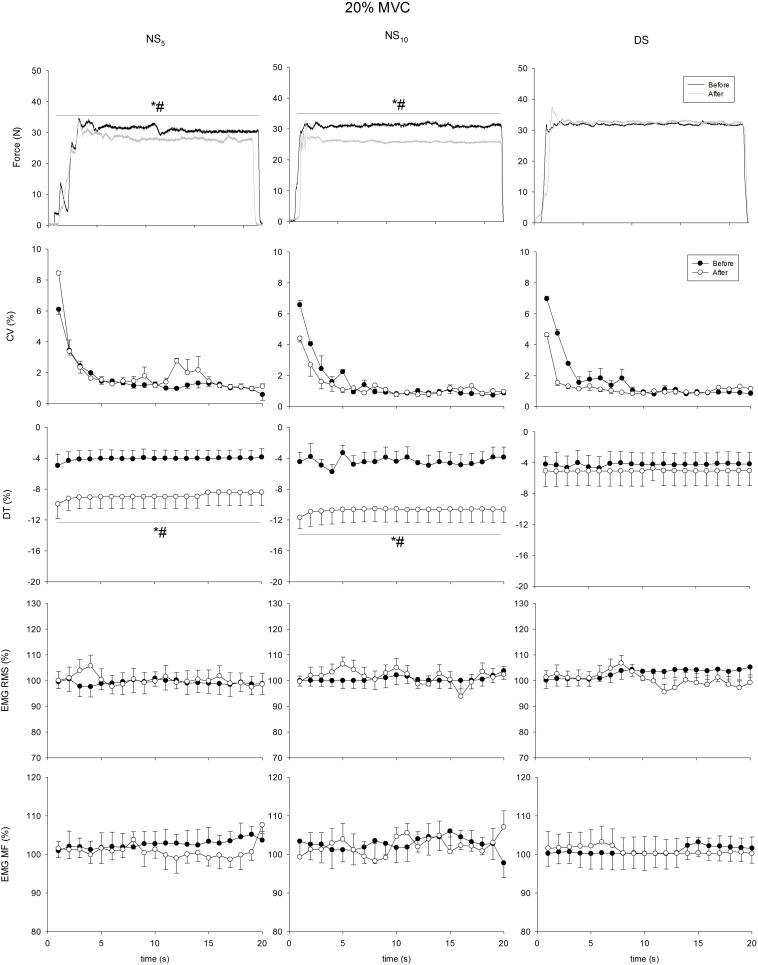
Coefficient of variation (CV), distance to target (DT), surface electromyogram root mean square (sEMG RMS), and mean frequency (sEMG MF) at 20% of maximum voluntary contraction (MVC) force maintained for 20 s at the beginning (pre, solid circle) and at the end (post, blank circle) of the shift cycle for the three groups. NS_5_, 5-day night shift; NS_10_, 10-day night shift; DS, day/swing shift. *p < 0.05 post vs. pre; ^#^*p* < 0.05 NS_5_ or NS_10_ vs. DS.

**FIGURE 5:**
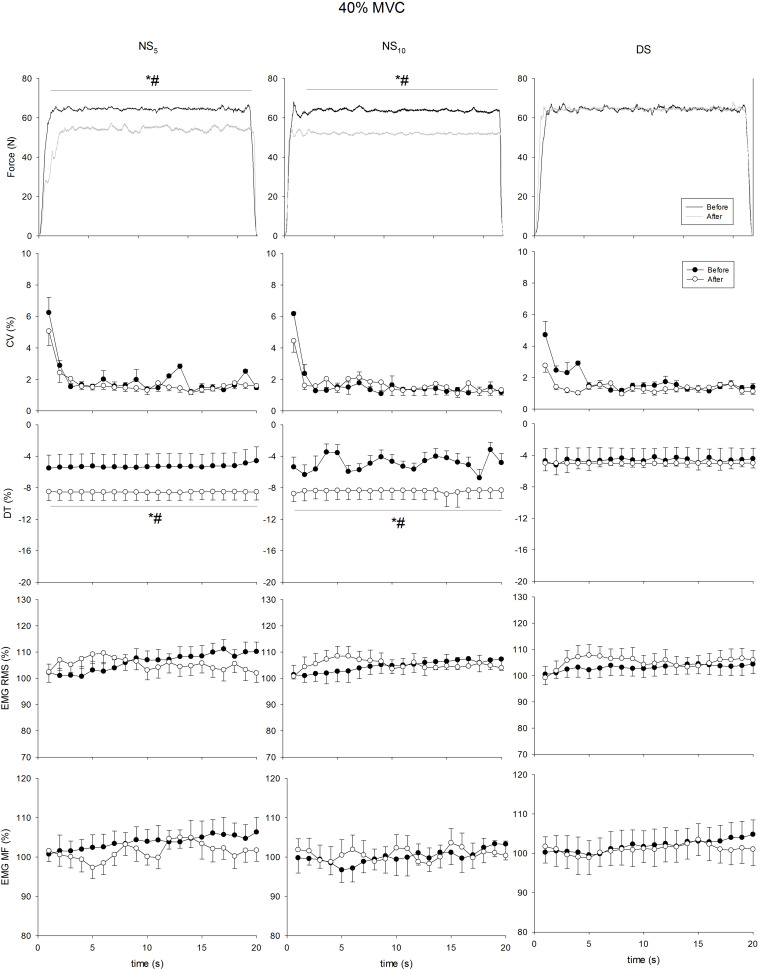
Coefficient of variation (CV), distance to target (DT), surface electromyogram root mean square (sEMG RMS), and mean frequency (sEMG MF) at 40% of maximum voluntary contraction (MVC) force maintained for 20 s at the beginning (pre, solid circle) and at the end (post, blank circle) of the shift cycle in the three groups. NS_5_, 5-day night shift; NS_10_, 10-day night shift; DS, day/swing shift. *p < 0.05 post vs. pre; ^#^*p* < 0.05 NS_5_ or NS_10_ vs. DS.

**FIGURE 6:**
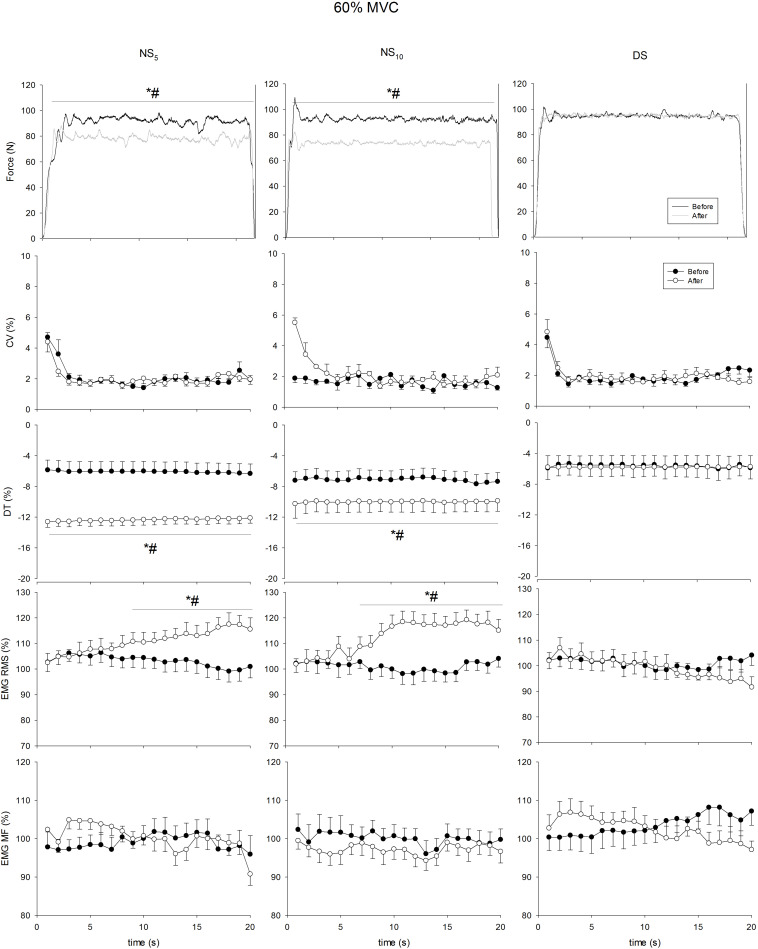
Coefficient of variation (CV), distance to target (DT), surface electromyogram root mean square (sEMG RMS), and mean frequency (sEMG MF) at 60% of maximum voluntary contraction (MVC) force maintained for 20 s at the beginning (pre, solid circle) and at the end (post, blank circle) of the shift cycle in the three groups. NS_5_, 5-day night shift; NS_10_, 10-day night shift; DS, day/swing shift. *p < 0.05 post vs. pre; ^#^*p* < 0.05 NS_5_ or NS_10_ vs. DS.

**FIGURE 7:**
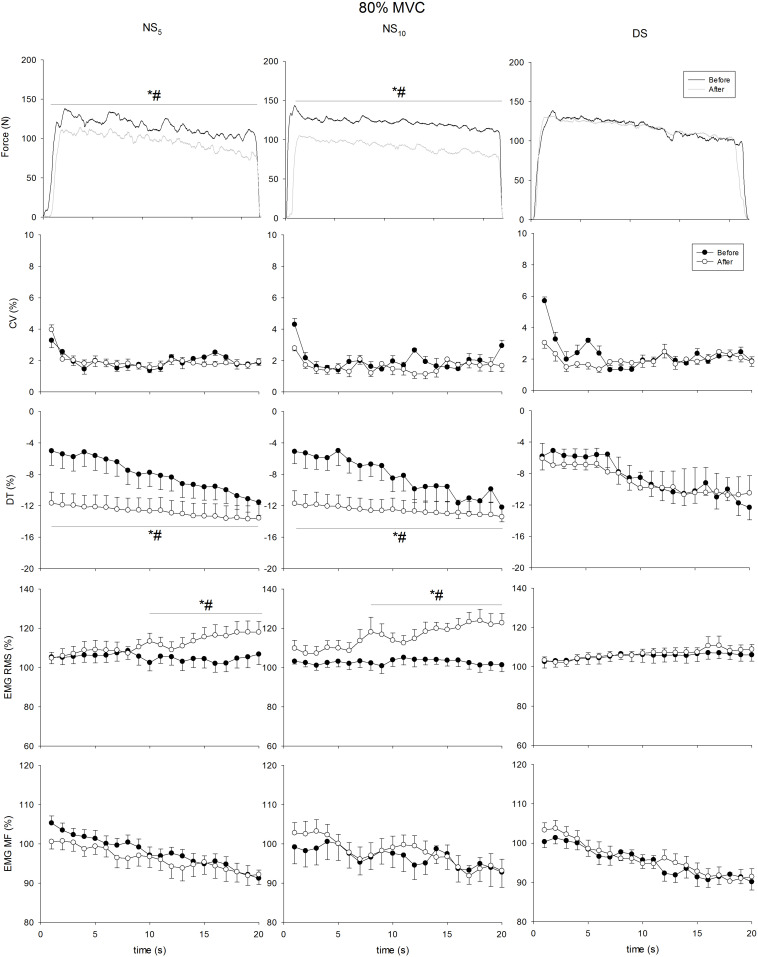
Coefficient of variation (CV), distance to target (DT), surface electromyogram root mean square (sEMG RMS), and mean frequency (sEMG MF) at 80% of maximum voluntary contraction (MVC) force maintained for 20 s at the beginning (pre, solid circle) and at the end (post, blank circle) of the shift cycle in the three groups. NS_5_, 5-day night shift; NS_10_, 10-day night shift; DS, day/swing shift. *p < 0.05 post vs. pre; ^#^*p* < 0.05 NS_5_ or NS_10_ vs. DS.

Figures 4–7 present the changes in force, CV, DT, EMG RMS and EMG MF for the groups on the submaximum contraction test. For all contractions, ANOVA disclosed a time × groups × contraction duration interaction in DT (*F* = from 5.35 to 12.77, *p* = from 0.007 to < 0.001) and sEMG RMS (*F* = 5.53, *p* = from 0.006 to < 0.001). Moreover, after an initial decrease in CV in all groups after the first 2 s (*p* < 0.001 for all comparisons), CV remained stable until the end of contraction, without differences in time and between groups. Unlike DS, DT significantly increased after the monitoring period in the NS_5_ and the NS_10_ group for all submaximum contractions [from + 30.7% (28.8/32.7) to + 44.1% (42.9/45.4), *d* from 2.73 (1.98/3.49) to 4.91 (3.86/5.95), from + 27.9% (26.6/29.2) to 42.3% (41.5/43.2), *d* from 1.83 (0.86/2.81) to 2.55 (1.45/3.65)]. *Post hoc* analysis revealed significant increases in sEMG RMS in the NS_5_ and the NS_10_ but not in the DS group after monitoring for contractions at 60% MVC force from the 9th and 8th s of contraction until the end of the task [+ 13.1% (8.4/18.2), *d* = 2.03 (1.35/2.73), + 19.7% (15.7/28.6), *d* = 3.11 (2.71/4.23)]. At 80% MVC force, the same pattern was found from the 12th s of contraction until the end [+ 12.9% (8.2/17.7), *d* = 2.01 (1.33/2.69), + 25.7% (18.7/32.7), *d* = 5.51 (3.74/7.27)]. No changes were observed for sEMG MF. In summary, CV and the sEMG MF over time remained similar among groups after the monitoring period; DT was reduced (force target underestimation) in NS_5_ and NS_10_ compared to DS; sEMG RMS behavior over time was higher during submaximal contraction at 60 and 80% MVC force.

**FIGURE 8:**
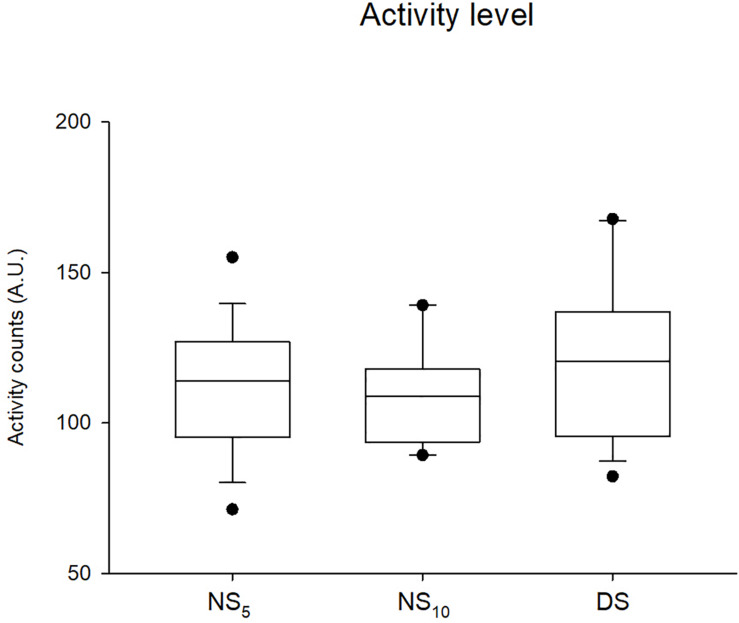
Actigraphic activity measured during the three shift cycles in the 5-day night shift (NS_5_), the 10-day night shift (NS_10_), and the day/swing shift (DS) group. Black dots represent the 95th/5th percentile.

**FIGURE 9:**
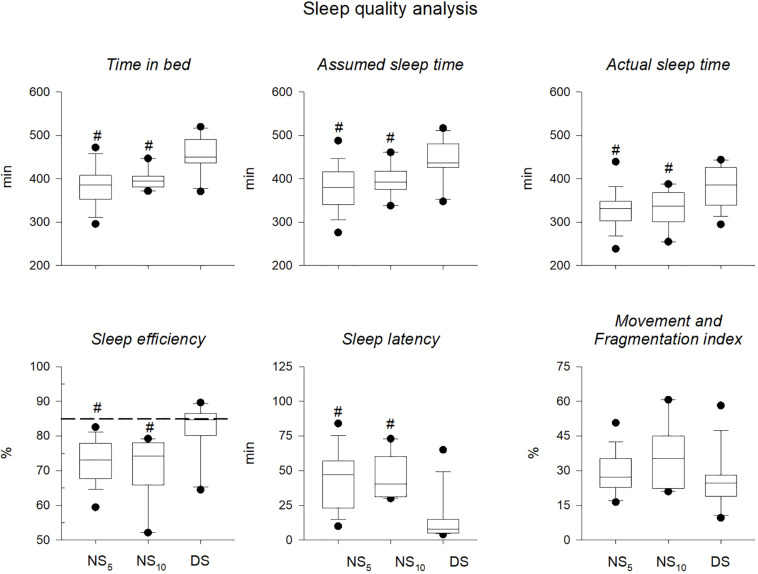
Sleep quality parameters. *Time in bed*, *Assumed sleep time, Actual sleep time, Sleep efficiency, Sleep latency*, and *Movement and fragmentation index* measured during the three shift cycles in the 5-day night shift (NS_5_), the 10-day night shift (NS_10_), and the day/swing shift (DS) group. ^#^*p* < 0.05 NS_5_ or NS_10_ vs. DS. Black dots represent the 95th/5th percentile. The dashed line in the Sleep efficiency panel represents 85%, which is defined a cut-off for an adequate sleep efficiency. An average of the entire monitoring period was calculated for each parameter and for each participant.

Daily activity levels and sleep parameters during the monitoring period. Activity levels were similar for the three groups (Figure 8). Except for the movement fragmentation index. Kruskal-Wallis test revealed significant differences between the NS_5_ and the NS_10_ vs. the DS group for time in bed [−71 min (−101/−41), *d* = −1.36 (−1.98/−0.74) and −55 min (−87/−23), *d* = −1.43 (−2.33/−0.52)], assumed sleep time [−63 min (−101/−24), *d* = −1.15 (−1.76/−0.55) and −47 min (−104/−10), *d* = −0.98 (−1.84/−0.12)], actual sleep time [−53 min (−87/−18), *d* = −1.08 (−1.68/−0.48), and −49 min (−100/−2), *d* = −1.04 (−1.90/−0.17)], sleep efficiency [−9.7% (−14.9/−4.5), *d* = −1.41 (−2.05/−0.78) and −11.2% (−18.8/−3.5), *d* = −1.32 (−2.23/−0.41)], and in sleep latency [+ 30 min (15/45), *d* = 1.37 (0.74/2.00) and + 30 min (7/52), *d* = 1.61 (0.66/2.55)] (Figure 9). ANOVA revealed a time x group interaction for CR−10 scale values (*F* = 3.26, *p* = 0.04). *Post hoc* analysis revealed no between−groups differences in CR−10 scale values before the monitoring period [NS_5_ = 4.0 (3.3/4.6); NS_10_ = 4.3 (2.9/5.6); DS = 3.9 (2.6/5.1)], while they significantly increased after the monitoring period in NS_5_ and NS_10_ but not in DS [NS5 = 5.3 (4.6/6.1), *d* = 0.86 (0.74/0.93); NS10 = 6.0 (5.1/6.9), d = 1.13 (0.89/1.27)].

Correlations. Table 2 presents the correlations between sleep quality parameters and the percentage difference in pre-post work-shift cycle for MVC force, sEMG RMS and MF. Except for the sleep latency, which presented a direct correlation, the time in bed, assumed sleep time, actual sleep time and sleep efficiency correlated inversely with the percentage difference in MVC force and sEMG RMS. Time in bed, sleep latency, and movement fragmentation index correlated also with the percentage difference in sEMG MF. Independently from sex, no correlations between age and the percentage changes in MVC force, EMG RMS, EMG MF, activity level and sleep quality parameters were found (*R* ranging from −0.201 to 0.041, *p* from 0.095 to 0.873).

**TABLE 2:** Correlations between sleep quality analysis parameters and percentage differences (D) pre-post shift cycle in maximum voluntary contraction (MVC) force of the finger flexor muscles, surface electromyography (sEMG) root mean square (RMS), and mean frequency (MF).

		Pre-post D MVC force	Pre-post D sEMG RMS	Pre-post D sEMG MF
Time in bed	*R, p*	−0.322, 0.007*	−0.464, < 0.001*	−0.386, 0.001*
Assumed sleep time	*R, p*	−0.334, 0.005*	−0.356, 0.003*	−0.226, 0.06
Actual sleep time	*R, p*	−0.362, 0.002*	−0.363, 0.002*	−0.232, 0.05
Sleep efficiency	*R, p*	−0.332, 0.005*	−0.392, 0.001*	−0.331, 0.005*
Sleep latency	*R, p*	0.372, 0.002*	0.340, 0.005*	0.222, 0.07
Movement and fragmentation index	*R, p*	0.162, 0.18	0.231, 0.05	0.251, 0.04*

*Correlations include all participants (n = 71). Cells report Pearson correlation coefficient (R) and significance level (p). ^∗^p < 0.05.*

## Discussion

With this study we investigated the effects of two different night shift schedules compared to only day/swing shift schedule on neuromuscular activation and muscle contraction during non-fatiguing and fatiguing contractions. The main findings demonstrated that, irrespective of the shift schedule, working night shifts: (i) affected both muscle maximum activation and maximum force generating capacity; (ii) reduced force accuracy when the muscle was involved in tasks requiring submaximum force output; and (iii) increased the occurrence of neuromuscular fatigue. All these findings are accompanied by an overall decrease in both sleep quantity and quality. Interestingly, the percentage reduction in muscle maximum activation and force correlated with the sleep quality parameters describing the total amount sleep time and its quality. Although the correlations reported here present a low (but significant) correlation coefficient, they indicate that the nurses reporting low sleep quality had the greatest decrease in force generation capacity. These aspects need to be considered when planning work schedules.

### Motor Control and Neuromuscular Fatigue

Our data show a clear reduction in maximum activation and muscle force output of the finger flexor muscles for two night shift groups compared to the day/swing shift group. Moreover, muscle function was correlated with sleep quality measured after different shift cycles. This reduction in MVC force suggests that working nights impaired maximum muscle activation and maximum force generating capacity, as demonstrated by the reduction in EMG RMS and EMG MF during MVC force after night shifts. During sustained contractions at 80% MVC force, a significant increase in EMG RMS was observed in the NS groups compared to the DS group. Such an increase is typically considered a marker of myoelectric fatigue manifestation (Merletti et al., 1990; Cè et al., 2019). Indeed, during sustained, high-intensity submaximum contraction, the muscle activation level increased over time to tentatively maintain a steady force output. An earlier and larger increase in the muscle activation level, as here reported, may indicate a major effort of the central nervous system to overcome the decrease in force output (Merletti et al., 1990; Cè et al., 2019).

Together with increased muscle fatigability, the present findings show a reduction in force accuracy (i.e., an underestimation of the force output with respect to the force target) irrespective of the force output level, which suggests difficulty to reach the force target and a reduction in neuromuscular control. Furthermore, this decline in neuromuscular control occurred also during low-intensity contraction that may best mimic the force intensity required for work-related tasks. This finding provides direct evidence for the previously reported increase in occupational injuries and health risks for both nurses and patients (Steele et al., 1999; Horwitz and McCall, 2004; Barger et al., 2005).

### Activity Level

One of the main issues in shift work is the difficulty to maintain an adequate level of daily activity, possibly predisposing to earlier onset of disorders typical of sedentarism (Matheson et al., 2014). There were no differences in the habitual activity level (i.e., IPAQ SF values) or the amount of activity recorded during the monitoring period between the three groups. Irrespective of the shift they habitually worked, about 87% of the participants classified as sufficiently active or active according to the IPAQ SF questionnaire responses. Taken together, these findings suggest that the shift work cycle did not prevent the nurses from keeping a sufficient level of activity.

### Sleep Parameters

The actigraphy data indicated a better quantity and quality of sleep in the DS compared to the NS_5_ and the NS_10_ group. These outcomes were further corroborated by the information provided by the CR-10 scale, which evidenced an increase in the perceived tiredness level in the NS groups, but not in DS group. The parameters of the amount of sleep (i.e., *time in bed*, *assumed sleep*, and *actual sleep time*) were higher for the DS than either NS group. Also, *sleep latency* (the amount of time required for sleep onset after retiring to bed) differed between the NS groups and the DS group, with a shorter length noted for the DS group. The parameter *sleep efficiency*, which measures the percentage of time in bed actually spent sleeping, was > 85% in the DS group and < 85% in the NS groups. This finding indicates that due to sleep disruption sleep quality was lower in the NS groups than in the DS group.

This finding indicates that the recovery strategies the nurses adopted after working the night shift were only partially efficient in promoting restoration and in limiting the effects of sleep disruption. Poorer sleep quality has been previously reported (Yuan et al., 2011; Booker et al., 2018; Min et al., 2019) and often associated with a higher risk of developing chronic fatigue (Yuan et al., 2011; Min et al., 2019) and other disorders including type 2 diabetes, cardiovascular disease, cancer (Roveda et al., 2017), and gastrointestinal disturbances (Smith and Eastman, 2012; Matheson et al., 2014). A reduction in sleep efficiency is a well-known consequence of night-shift work, possibly because sleep duration depends on the circadian phase at which it occurs, where the longest sleep duration and the best sleep efficiency are obtained when sleep begins when core body temperature is lowest (Wyatt et al., 1999). This usually occurs a few hours before waking up. Since the circadian clock of most night shifters does not align with daytime sleep (Folkard, 2008), their minimum temperature usually remains at the night-time (working hour) level, and the duration of daytime sleep is shorter because the circadian clock promotes wakefulness. Moreover, circadian secretion of melatonin and cortisol are also altered after working several night shifts, with a continuous peak in the early morning hours and at night of melatonin and cortisol, respectively (Boivin and Boudreau, 2014). Moreover, sleep disruption due to extended night shift work could be related to increased rates of occupational errors and attentional failures (Patterson et al., 2010).

### Overall Considerations

Altogether, the decrease in MVC force and EMG activity, the increase in neuromuscular fatigue, and the reduction in muscle force accuracy may be interpreted as different markers that reflect the same scenario: impaired muscle performance ascribable to a disruption in the sleep-wake cycle, which is a common complaint in night-shift workers. Sleep-wake cycle disruption leads to a decrease in sleep quantity and quality, resulting in acute and/or chronic fatigue often accompanied by impaired neuromuscular control (Yuan et al., 2011; Min et al., 2019). The correlations between MVC force and muscle activation level and sleep parameters suggest a link between reduced muscle performance and lower overall sleep quality.

Although the present study does not identify the exact mechanism/s underlying the observed decrease in muscle performance after night shift work, a hypothesis for the difference between the NS and the DS groups may be advanced nonetheless. A variety of neuroendocrine mechanisms may be cited to explain the occurrence of reduced maximal muscle activation, force generating capacity and altered neuromuscular control in night shift workers, including (i) impairment in circadian secretion of melatonin and cortisol and in regulation of core body temperature (Boivin and Boudreau, 2014); (ii) momentary reduction in energy resources, such as glycogen (Dalsgaard and Secher, 2007), depletion of synaptic vesicles/calcium (O’Donovan and Rinzel, 1997) or accumulation of adenosine (Brundege and Dunwiddie, 1998; Brambilla et al., 2005; Lovatt et al., 2012), which can impair cross-bridges formation; (iii) possible alteration of the noradrenergic and other neuromodulatory systems (Leonard et al., 1987; Bramham and Srebro, 1989), thus reducing the ability of the brain to promptly respond to external inputs by changing the activity pattern in prefrontal cortex and possibly altering motor control (Sara, 2015); (iv) impairment of the adenosinergic/dopaminergic mechanisms at the level of the corpus striatum (which coordinates multiple aspects of motor and action planning, decision making, motivation, reinforcement, and reward perception) (Satterfield et al., 2017); and (v) earlier fatigability of the locus coeruleus neurons targeting the prefrontal cortex and underlying the cognitive impairment associated with sleep deprivation, which can possibly affect the motor control (Faw, 2003).

The present study comes with some acknowledged limitations. Firstly, the combined analysis of the EMG and force signals represented a good compromise between (i) providing non-invasively objective and physiological parameters to detect possible night shift-induced impairments in neuromuscular activation and force generating capacity, and (ii) having an approach that would be feasible in an ecological context and compatible with a hospital environment. However, this approach did not permit the discrimination of the exact mechanism/s involved in the reduction of neuromuscular activation and motor control in night shift nurses. Secondly, EMG data should be used with great caution when making assumptions about motor control strategies at a motor unit level (Del Vecchio et al., 2017). Thirdly, information about the peri- and post-menopausal condition of the nurses would have helped to control for a possible bias introduced by the association between menopause and sleep disorders. However, in the light of similar values of the CR-10 scale for the perceived tiredness retrieved between groups before the monitoring period, and the lack of correlations observed here between age, sleep quality variables, changes in muscle force generating capacity, and force control, it is likely that this possible bias had only a limited effect on the overall findings reported in the present investigation. Fourthly, the present experimental set-up did not permit to clearly distinguish the effect of the previous night of sleep deprivation form the cumulative sleep-wake disruption as a result of the entire monitoring period. Lastly, the different day time at which neuromuscular evaluations were performed could have influenced the results. However, the evaluations coincided with the end of either the night-shift cycle (i.e., at 7.00 a.m.) or with the end of one of the diurnal cycles (i.e., at 2.00 or 9.00 p.m.) according to the tested group. Likely, testing the participants at the same day time (i.e., at 7.00 a.m.) would have masked the fatiguing effect induced by the shift, possibly reinforcing the already observed inter-group differences in terms of force generating-capacity reduction.

## Conclusion

In conclusion, our findings clearly demonstrate an impairment in neuromuscular function in both NS groups independent of their shift cycle schedule. The neuromuscular alterations, i.e., reduction in maximum muscle activation level, muscle force output of the finger flexor muscles, earlier and greater myoelectric fatigue manifestation, and lower muscle accuracy, were accompanied by poorer sleep quantity and quality. Nonetheless, the NS groups were able to maintain an adequate level of physical activity, as measured by IPAQ responses. These findings may inform recommendations to improve recovery strategies that can counteract neuromuscular alterations in nursing staff working the night shift.

## Data Availability Statement

The datasets generated for this study are available on request to the corresponding author.

## Ethics Statement

The studies involving human participants were reviewed and approved by the Ethical Committee of San Raffaele Hospital (CE: 156/int/2017). The patients/participants provided their written informed consent to participate in this study.

## Author Contributions

EC, ER, AMo, PD’A, GB, and FE conceived and designed the study. EC, ER, LG, LC, and AMu performed the experiments. EC, LG, LC, and AMu analyzed the data. EC, CD, ER, AMo, LG, LC, and AMu interpreted the results. EC, CD, LG, LC, AMu, and FE prepared the figures. EC, CD, and FE drafted, edited, and revised the manuscript. All authors contributed to the article and approved the submitted version.

## Conflict of Interest

The authors declare that the research was conducted in the absence of any commercial or financial relationships that could be construed as a potential conflict of interest.

## Abbreviations

CV: coefficient of variation
DS: day/swing shift
DT: distance to target
IPAQ: international physical activity questionnaire
MF: mean frequency
MVC: maximum voluntary contraction
NS: night shift
RMS: root mean square
sEMG: surface electromyography.
